# The efficacy of the novel “wall pierce” technique for common femoral access in endovascular therapy

**DOI:** 10.1002/ccr3.9233

**Published:** 2024-07-31

**Authors:** Shinsuke Mori, Norihiro Kobayashi, Masakazu Tsutsumi, Yoshiaki Ito

**Affiliations:** ^1^ Department of Cardiology Saiseikai Yokohama City Eastern Hospital Kanagawa Japan

**Keywords:** common femoral artery, endovascular therapy, puncture, trans femoral approach, wall PIERCE technique

## Abstract

Under challenging common femoral artery access scenarios, the “wall PIERCE” technique, which utilizes a larger puncture needle to pierce the vessel wall along the guidewire, facilitates sheath insertion. This method proved successful in two cases without any complications, presenting a valuable addition to strategies for addressing challenging sheath insertion scenarios.

## 1 INTRODUCTION

With the evolution of devices, medical procedures involving less invasive approaches, such as transradial access, distal radial access, and tibiopedal arterial (tibiopedal arterial minimally invasive retrograde revascularization) access are preferred for percutaneous coronary intervention (PCI) and endovascular therapy (EVT).^1^, ^2^, ^3^ However, the common femoral artery (CFA) approach remains necessary for procedures requiring large‐diameter sheath, such as an 8Fr for PCI or EVT below the inguinal region. During this approach, even after a successful access and insertion of a 0.035‐inch guidewire, inserting the sheath can sometimes be difficult. This is particularly common in cases of severe calcification of the CFA vessel wall, having a history of multiple CFA approaches to chronic limb‐threatening ischemia, or accessing the CFA after endarterectomy. Typically, these challenges are addressed by first using a bougie with a dilator attached to the sheath, replacing the guidewire with a sturdier 0.035‐inch guidewire, or replacing the sheath with a smaller diameter sheath. If sheath insertion remains difficult, the puncture or approach site should be changed.

We have recently encountered two cases wherein a sheath was successfully inserted using the “wall percutaneous direct needle puncture of calcified plaque (PIERCE)” technique. This technique involves using a larger puncture needle to pierce the vessel wall along the guidewire. In this case series, we present these two cases.

## 2 CASE 1

### 2.1 Case history and examination

**FIGURE 1:**
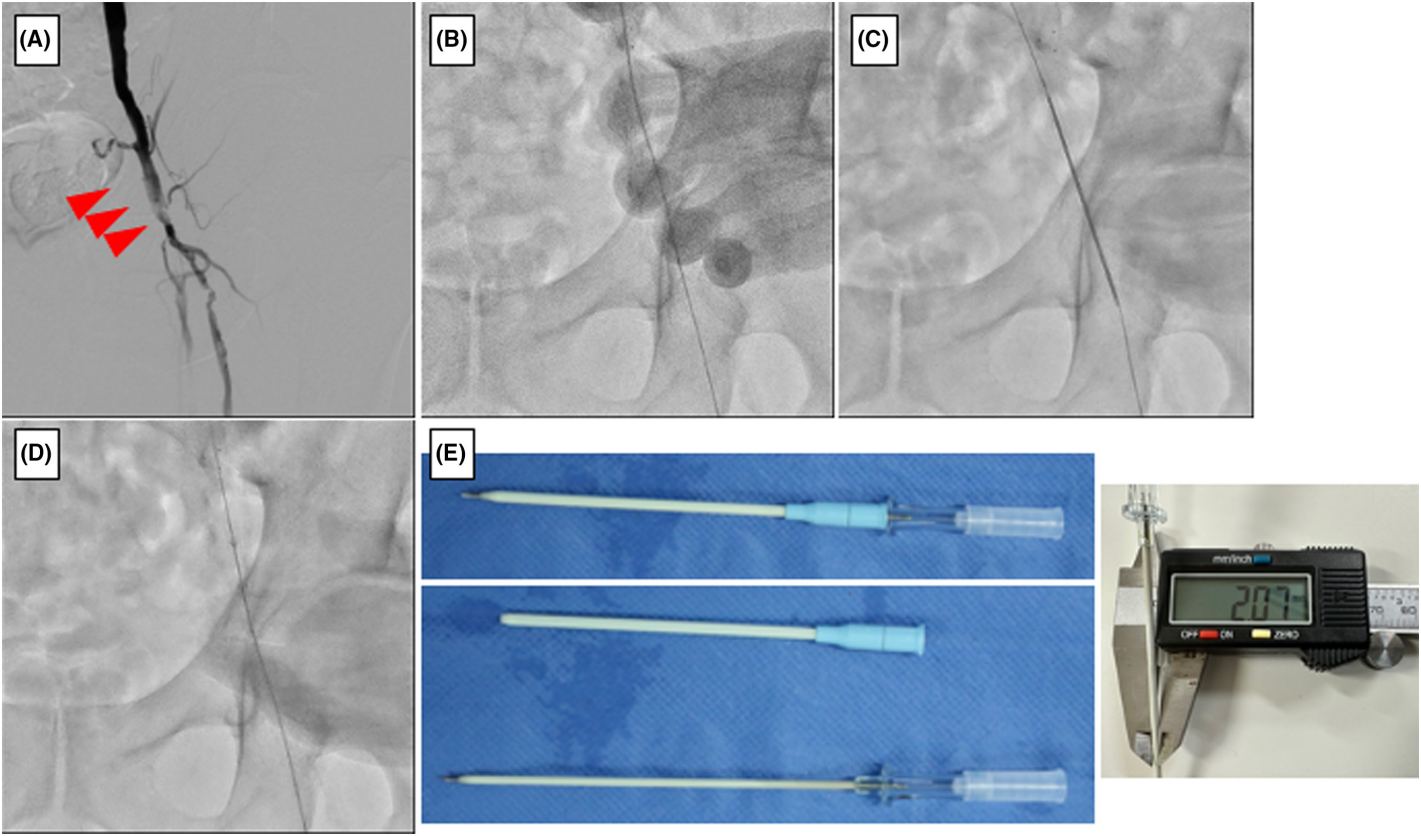
**(**A) Left CFA lesion before endarterectomy (arrow indicates calcification) (B) A 0.035‐inch guidewire insertion after the CFA puncture. (C) The “wall PIERCE” technique. (D) Successful insertion of a 6Fr sheath. (E) BD AngiocathTM IV Catheter 12GA × 3.00IN (Left figure). The thickness of the needle as measured with a caliper was 2.07 mm.

A 60‐year‐old male patient presented with Rutherford class 5 lower‐extremity artery disease with resting pain and an ischemic ulcer on his left fourth toe. He had a history of diabetes mellitus and was on hemodialysis. A few years prior, he underwent an endarterectomy for a calcified lesion in the left CFA (Figure 1A). Duplex ultrasonography revealed chronic total occlusion of the left distal superficial femoral artery.

### 2.2 Differential diagnosis, investigations, and treatment

We opted for an ipsilateral approach from the left CFA and accessed it using an 18GA puncture needle. The insertion was performed successfully; a 0.035‐inch guidewire was advanced into the superficial femoral artery. However, the sheath could not follow the guidewire owing to the hardened vessel wall resulting from the prior endarterectomy of the CFA (Figure 1B). To overcome this challenge, we employed the “wall PIERCE” technique, which involves inserting a 12GA puncture needle (BD Angiocath™ IV Catheter 12GA × 3.00IN; BD Bioscience, Franklin Lakes, NJ, USA) along the guidewire to penetrate the CFA vessel wall, thereby facilitating the subsequent passage of the sheath (Figure 1C). After performing the “wall PIERCE” technique, the sheath was successfully inserted (Figure 1D). Figure 1E shows the BD Angiocath™ IV Catheter 12GA × 3.00IN (left upper panel). A puncture needle without a catheter was used for the wall PIERCE technique (left, lower panel).

## 3 CASE 2

### 3.1 Case history and examination

A female patient in her 70s with Rutherford class 5 lower‐extremity artery disease presented with resting pain and gangrene in her left third and fifth toes. She had a history of diabetes mellitus and was on hemodialysis.

### 3.2 Differential diagnosis, investigations, and treatment

**FIGURE 2:**
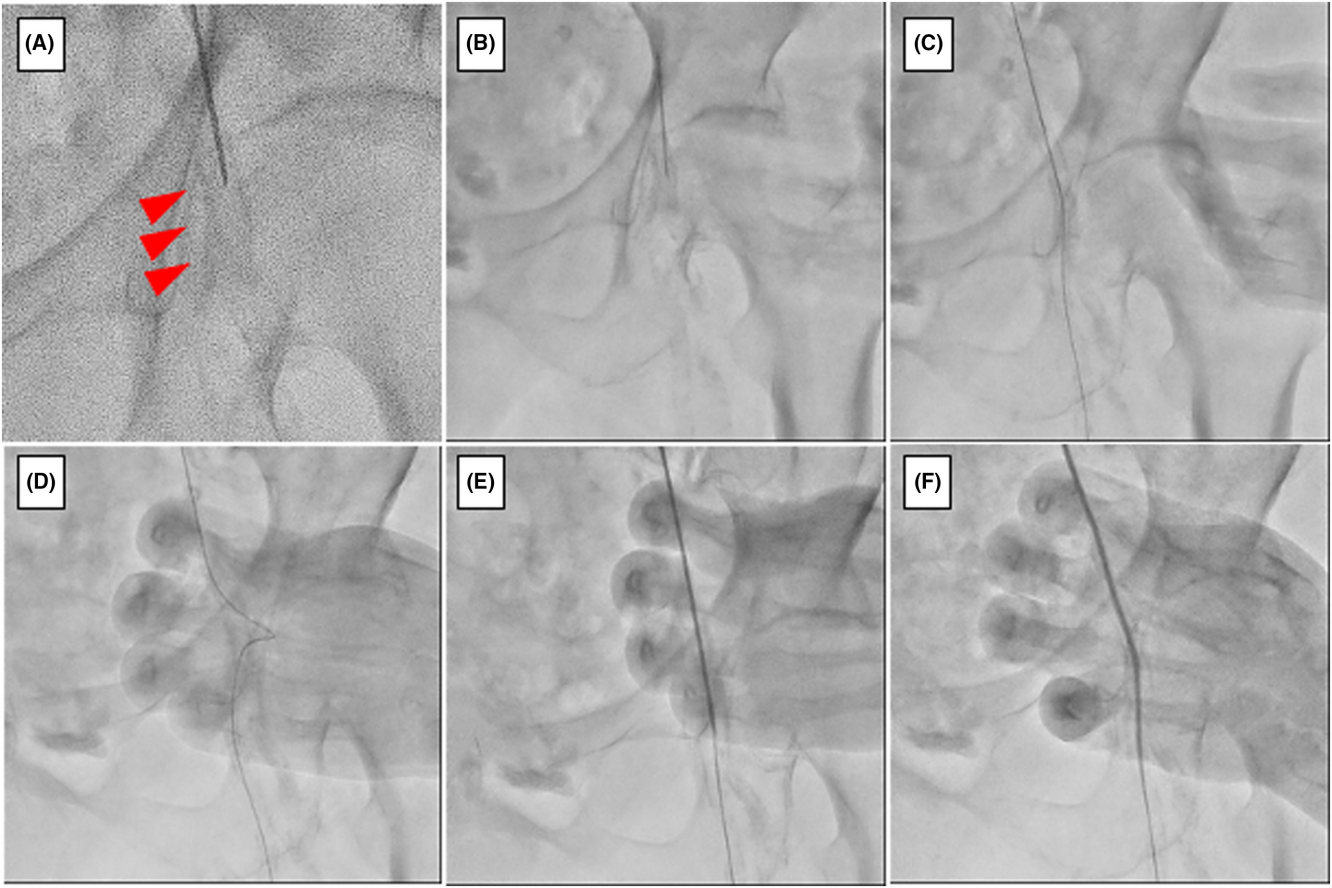
(A) Severe calcification is observed in the left CFA under fluoroscopy. (B) CFA puncture with an 18GA needle. (C) A 0.035‐inch guidewire insertion. (D) Failure of 5Fr sheath insertion. Note that the guidewire is bent. (E) The “wall pierce” technique. (F) Successful insertion of 5Fr sheath.

We opted for an ipsilateral approach from the left CFA and performed a puncture with an 18 GA puncture needle. Fluorography revealed severe calcification at the insertion site in the left CFA (Figure 2A). The artery was successfully accessed; a 0.035‐inch guidewire was advanced into the superficial femoral artery. However, the sheath could not follow the guidewire as the vessel wall was hardened owing to severe calcification (Figure 2B–D). Therefore, the “wall PIERCE” technique was used to penetrate the CFA vessel wall, thereby facilitating the subsequent passage of the sheath (Figure 2E). After performing the “wall PIERCE” technique, the sheath was successfully inserted (Figure 2F).

## 4 DISCUSSION

Under some scenarios, transfemoral access is selected when performing the PCI or EVT procedures. However, dense calcifications at the puncture site or blood vessel wall stiffening after endarterectomy could hinder sheath advancements, even if the puncture needle and guidewire were successfully inserted. We suggest, based on our experience, that the “wall PIERCE” technique could be effective in such cases.

Nakama et al. reported an inner PIERCE technique for advancing through hard lesions such as calcifications.^4^ This method involves modifying a highly calcified lesion using a retrograde long puncture needle when the guidewire is passed through the infrapopliteal lesion and a pull‐through is achieved. However, the balloon does not pass through. This modification is highly effective and allows the balloon to pass through, facilitating balloon expansion. We applied this technique at the CFA puncture site. Performing this piercing technique, a 12GA needle is used, which creates a hole equivalent to 2 mm around the guidewire (Figure 1E, right panel), effectively accommodating a 4Fr sheath. Thus, piercing the blood vessel wall with a 12GA needle facilitates subsequent sheath insertion. In addition, even if a 5Fr sheath is inserted, there is no leakage around the sheath during EVT. Another advantage of this technique is the cost‐effectiveness of the puncture needle.

However, the needle is relatively bulky. Therefore, changing the guidewire to a 0.035‐inch support‐type guidewire to prevent potential injury to the guidewire by the needle and its subsequent rupture is recommended. Notably, at our hospital, we used the “wall PIERCE” technique in nine cases between January 2021 and January 2024, and sheath insertion was successful in all cases, without complications such as guidewire rupture or hematoma (Table 1).

**TABLE 1:** Patient list.

No.	Sex	Age	Side	Hemodialysis	CLTI	Sheath size	Direction	Success	Complication	Reason for wall pierce
1	Male	59	Left	+	+	6Fr	Downwards	Yes	No	Post‐endarterectomy
2	Female	77	Left	+	+	5Fr	Downwards	Yes	No	Severe calcification
3	Female	87	Left	−	+	6Fr	Downwards	Yes	No	Severe calcification
4	Male	73	Right	+	+	5Fr	Downwards	Yes	No	Multiple punctures
5	Female	96	Right	−	+	5Fr	Downwards	Yes	No	Severe calcification
6*	Male	66	Left	+	+	6Fr	Downwards	Yes	No	Severe calcification
7*	Male	68	Left	+	+	7Fr	Downwards	Yes	No	Severe calcification
8*	Male	68	Left	+	+	5Fr	Downwards	Yes	No	Severe calcification
9*	Male	68	Left	+	+	5Fr	Downwards	Yes	No	Severe calcification

Abbreviation: CLTI, chronic limb‐threatening ischemia.

* The same patient.

The “wall PIERCE” technique has been mainly used only in EVT cases. However, we suggest that it can also be utilized when inserting a PCI sheath. Although we exclusively used the BD AngiocathTM IV Catheter 12GA x 3.00IN, the same procedure could be performed with other needles close to 12GA in size.

## 5 CONCLUSION

The “wall PIERCE” technique could be a valuable addition to strategies for addressing difficult sheath insertion, potentially eliminating the need for changing the puncture or approach site.

## AUTHOR CONTRIBUTIONS

**Shinsuke Mori:** Conceptualization; writing – original draft. **Norihiro Kobayashi:** Supervision. **Masakazu Tsutsumi:** Conceptualization; data curation. **Yoshiaki Ito:** Supervision.

## FUNDING INFORMATION

The authors received no financial support for the research, authorship, or publication of this manuscript.

## CONFLICT OF INTEREST STATEMENT

S.M. received speaker's fees from BD and Terumo. M.T. received speaker's fee from Abbott. Y.I. received speaker's fees from Abbott, Terumo, Boston, Medtronic, and NIPRO. The remaining author declares no conflict of interest.

## ETHICS STATEMENT

The study protocol was conducted following the tenets of the Declaration of Helsinki and was approved by the local ethics committee of our hospital.

## CONSENT

Written informed consent was obtained from all patients.

## Data Availability

Data and materials cannot be shared openly to protect the patient's privacy.

## References

[1] Ferrante G , Rao SV , Jüni P , et al. Radial versus femoral access for coronary interventions across the entire spectrum of patients with coronary artery disease: a meta analysis of randomized trials. JACC Cardiovasc Interv. 2016;9(14):1419‐1434. doi:10.1016/j.jcin.2016.04.014 27372195

[2] Kiemeneij F . Left distal transradial access in the anatomical snuffbox for coronary angiography (ldTRA) and intervention (ldTRI). EuroIntervention. 2017;13(7):851‐857. doi:10.4244/EIJ-D-17-00079 28506941

[3] Mustapha JA , Saab F , McGoff T , et al. Tibio‐pedal arterial minimally invasive retrograde revascularization in patients with advanced peripheral vascular disease: the TAMI technique, original case series. Catheter Cardiovasc Interv. 2014;83(6):987‐994. doi:10.1002/ccd.25227 24214522

[4] Nakama T , Muraishi M , Obunai K , Watanabe H . Efficacy of the novel inner PIERCE technique for severely calcified below‐the‐knee occlusions in a patient with chronic limb‐threatening ischemia. Catheter Cardiovasc Interv. 2020;96(6):1317‐1322. doi:10.1002/ccd.29255 32930477

